# Comparison of Adhesive Properties of Polyurethane Adhesive System and Wood-plastic Composites with Different Polymers after Mechanical, Chemical and Physical Surface Treatment

**DOI:** 10.3390/polym11030397

**Published:** 2019-03-01

**Authors:** Barbora Nečasová, Pavel Liška, Jakub Kelar, Jiří Šlanhof

**Affiliations:** 1 Faculty of Civil Engineering, Brno University of Technology, Veveří 331/95, 602 00 Brno, Czech Republic; liska.p@fce.vutbr.cz (P.L.); slanhof.j@fce.vutbr.cz (J.S.); 2 Faculty of Science, Masaryk University, Kotlářská 2, 611 37 Brno, Czech Republic; jakub.kelar@mail.muni.cz

**Keywords:** adhesion, adhesive, bond, cohesion, composite, joint, multi-hollow surface dielectric barrier discharge plasma source (MHSDBD), sandpaper, wood-plastic composite (WPC)

## Abstract

The cost of most primary materials is increasing, therefore, finding innovative solutions for the re-use of residual waste has become a topic discussed more intensely in recent years. WPCs certainly meet some of these demands. The presented study is focused on an experimental analysis of the effect of surface treatment on the adhesive properties of selected WPCs. Bonding of polymer-based materials is a rather complicated phenomenon and modification of the bonded area in order to improve the adhesive properties is required. Two traditional types of surface treatments and one entirely new approach have been used: mechanical with sandpaper, chemical with 10 wt % NaOH solution and physical modification of the surface by means of a MHSDBD plasma source. For comparison purposes, two high-density polyethylene based products and one polyvinyl-chloride based product with different component ratios were tested. A bonded joint was made using a moisture-curing permanently elastic one-component polyurethane pre-polymer adhesive. Standardized tensile and shear test methods were performed after surface treatment. All tested surface treatments resulted in an improvement of adhesive properties and an increase in bond strength, however, the MHSDBD plasma treatment was proven to be a more suitable surface modification for all selected WPCs.

## 1. Introduction

Wood-plastic composites (known as WPCs) belong to the category of fiber-reinforced composite materials [1]. They combine the stability of wood fibers with the durability of synthetic thermoplastic polymers, particularly PE, PP and PVC [2,3,4,5,6]. This combination allows a wide range of applications, while also offering the option of using the waste products of the forestry and wood industries as well as some types of recycled plastic waste [3,4,5,6,7,8]. Even though the technology appeared almost 100 years ago [5,9], the greatest success of production and demand for these products has only been seen in recent years [5,10,11,12]. The statistics show that the major sector in which WPCs are applied is the construction industry, with a 76% share [8,11]. WPCs find uses primarily as flooring, fencing and façade cladding [11,12,13,14].

The presented study is focused on an experimental analysis of the effects of surface treatment on the adhesive properties of selected WPC façade cladding. Currently, established traditional joining methods, e.g., rivets or screws, are commonly used, however, as some recent studies indicate [12,15,16,17] these methods cause higher failure rates. The main reason can be seen as the high stress concentration at the joint, which results in the occurrence of fatigue-caused cracks [16,17]. Welded or adhesive-bonded joints have proven to be more suitable and durable alternatives [4,10,15,18,19,20,21]. They ensure better stress distribution in the joint that consequently results in greater construction stiffness and greater stress resistance [22,23,24]. Unfortunately, the bonding of WPCs is more complicated than the bonding of traditional materials [2,4,18]. This statement was also confirmed by authors in previous research, where the adhesive properties of solid timber, cement-bonded particle board and WPC were studied [25]. The bondability of WPCs is highly affected by the thermoplastic component of the product, i.e., its thermoplastic matrix, which has a very low surface energy and bad wettability properties [1,2,11,26,27]. The major prerequisite for perfect adhesion is the creation of interaction forces [23,24,26,27]. It has been settled that the higher the value of surface stress the higher the polarity of the given surface. Therefore, it is advisable to use an adhesive with lower polarity than that of the adherend. This allows the wetting of the adherend contact surface. However, in façade systems, this step is greatly complicated by the limited range and number of products. At present, less than eight certified adhesive systems are available on the market. Moreover, there are only polyurethane or modified polymer based adhesives. Both types have rather poor wetting characteristics in combination with plastics. The wrong combination of materials of a façade system may result in considerably shorter service life and it can significantly affect the bondability and adhesive properties. As a result, it is advisable to increase surface polarity by modifying the bonded areas.

As proven by selected case studies, the bonding of WPCs is almost impossible without prior surface modification [11,17,18,19,20,21,26,28] and also without the application of primer or any promoting agent. Using primer puts greater demands on the cleanness of the work environment and prolongs the installation time, therefore, a surface treatment which would allow for the promoting agent to be eliminated is desired. According to some authors [11,22], WPC surface modifications can be divided into three basic categories: mechanical modification (i.e., sandblasting and roughening), chemical modification (i.e., application of acid or alkaline solutions as, for example, chromic acid, sodium hydroxide or fluorine) and physical modification (i.e., LP/AP plasma, corona, flame or laser). It is believed that the selection of the appropriate method depends on the matrix material and the used WPC formulation [11,18,19]. For this reason, three different WPC façade claddings with dissimilar formulations, polymer matrix and wood flour were selected in this study to verify the effect and versatility of surface modifications. 

In this paper the effect of three different surface treatment methods on the improvement of the adhesive properties of WPCs was studied: Mechanical with sandpaper, chemical with a 10 wt % NaOH solution and physical modification of the surface by means of a Multi-Hollow Surface Dielectric Barrier Discharge plasma source (MHSDBD). The first selected surface treatment is the mechanical modification using P40 grid sandpaper. According to Kraus et al. [11] and Oporto et al. [18] joining of WPC products is usually performed after brushing or sawing. It is a very common, economical and undemanding preparation process that allows for the more prominent appearance of wood fibers on the surface. This modification regularly shows very good wetting results and strength increases. The sandpaper coarseness was determined based on previous experience modifying cement-based composites and WPC [25,29,30]. P80 and P240 sandpaper was used in previous research cases, both types are less abrasive and leaves a finer surface. Even though the final results were very promising and a 100% increase in shear strength was monitored, a visual inspection showed that the surface was not sufficiently modified, therefore, a rougher coarseness was tested here. The chemical alkali treatment is the next of the selected surface treatments. Application of a 10 wt % sodium hydroxide was chosen due to its common availability, low cost and simple application process. Moreover, the concentration used has been shown to work well with different types of materials, especially with wood fibers [11,31,32]. Agarwal et al. [33] monitored a 120% increase in impact strength, if wood fibers in WPC were treated with a 1 wt % and 3 wt % NaOH solution. The last selected surface treatment was physical modification performed with a Multi-Hollow Surface Dielectric Barrier Discharge plasma source (MHSDBD). Plasma treatment is one of the most versatile surface treatment techniques, it is considered the most effective, sustainable and low-cost method for surface treatment of polymer-based materials compared with traditional treatments [27]. The positive effect of plasma treatment has already been proven in combination with different formulations of WPCs. Kraus et al. [11] investigated and compared LP and AP plasma surface treatments of PP-based WPC, Wolkenhauer et al. [26] studied the potential of dielectric barrier discharge (DBD) at atmospheric pressure and ambient air in combination with PP- and PE-based WPCs. Surfaces of WPCs formulated with HDPE and PP were treated and the tensile bond strength increased 5 times after DBD plasma treatment. The MHSDBD also generates atmospheric-pressure plasma in ambient air, however, it is the result of a combination of two methods of obtaining non-isothermal plasma at atmospheric pressure; gas flow and dielectric insertion into the discharge space. One of the advantages of MHSDBD is the possibility of driving it under atmospheric pressure at low power. The MHSDBD plasma has been adapted for the modification of small planar objects and for the need for patterned surface treatment [34,35]. This is the first application of this plasma source in combination with WPCs. However, in a case study from 2016, a similar plasma source, diffuse coplanar surface barrier discharge (DCSBD), was used to improve the adhesive properties of WPC formulated with 50% HDPE. The obtained results showed a 100% increase in the bond shear strength, and a 30% change in the failure mode, from adhesive to cohesive, was observed [29]. MHSDBD is in many ways similar to the industrially available DCSBD plasma source. This similarity is in its construction, i.e., used materials and geometry of electrode system, and physical parameters such as temperature or plasma power density. However, MHSDBD has proven to be much more effective in the treatment of topologically challenging materials thanks to its higher effective thickness plasma layer. Since the main parameters such as electrode geometry, plasma temperature and plasma power density are similar to DCSBD, we have been able to suggest the same treatment times, as in our previous work with DCSBD in 2016. The DCSBD plasma source was not suitable for WPC surface modification since the surfaces of the selected materials have an embossment which imitates the look of real wood, the depth of the embossment varied for each tested WPC and ranged between 0.05–0.5 mm. 

The focus of this study is assessing the surface treatment effectiveness. The main aim is to determine whether the selected plasma treatment can be used for different types of WPCs with the same or better results compared to more traditional methods, i.e., mechanical and chemical treatments. The effect is investigated on bonded assemblies of three types of WPCs in combination with a moisture-curing permanently elastic one-component polyurethane adhesive. The adhesive is a part of one of the most common systems intended for façade applications [35,36]. The shear strength of a single lap joint under tensile stress and adhesion of bonded joint under axial tensile stress were measured with a tensile test, to verify the impact of the selected surface treatment methods.

## 2. Materials and Methods

### 2.1. Materials Selection

The adhesive system used in this study is produced by Dinol GmbH and is intended only for façade bonding. It consists of the moisture-curing permanently elastic one-component polyurethane pre-polymer adhesive Dinitrol F500LP Polyflex, the primer Dinitrol Multiprimer 550 that acts as a bonding promoter for the surfaces of adherends, and Dinitrol 520 Cleaner that degreases the bonded surfaces. To ensure durable and efficient joints, all components have to be used as recommended by the manufacturer. According to the information provided in the technical data sheet, Dinitrol F500LP Polyflex has a tensile strength of 9.0 MPa with a maximum elongation at break of 600% and a shear strength after 7 days of 5.5 MPa [37].

**Figure 1:**
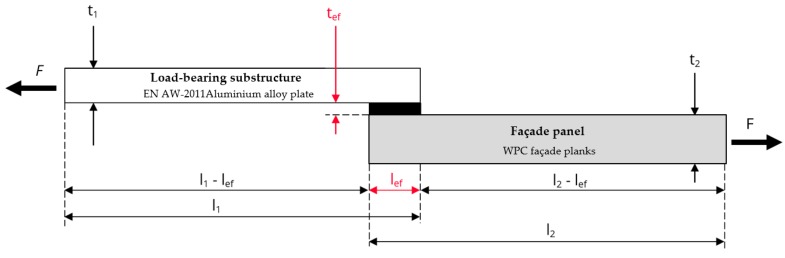
Single lap joint test sample geometry—cross section.

**Figure 2:**
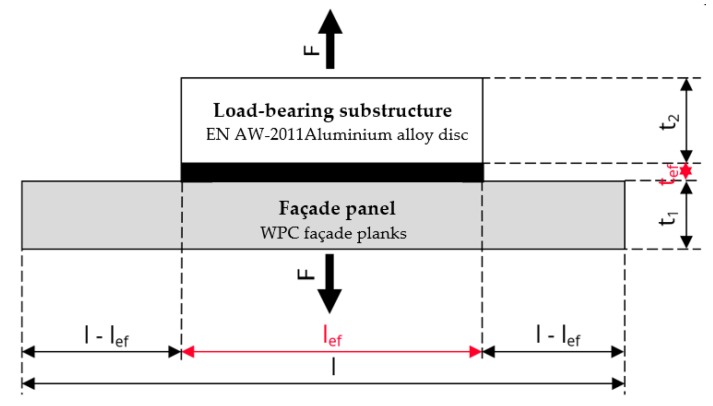
Adhesion test sample assembly—cross section.

The material representing the load-bearing substructure was selected with the intent to avoid the premature failure of joints due to their incompatibility with the chosen adhesive system. The EN AW-2011 aluminium alloy with a tensile strength of 295.0 MPa and a yield strength of 195.0 MPa was chosen. The thickness of the material selected for the shear strength test was 5 mm, see Figure 1; the samples used in adhesion testing were 15 mm thick, see Figure 2.

The first tested WPC cladding (referred to as WPC_45/45 in the text) was 21 mm thick. The ratio of components in the tested material was 45 wt % beech wood flour, 45 wt % PVC (specifically VYNOVATM S6502 high molecular weight and high porosity vinyl chloride homopolymer) and 10 wt % additives [38]. A material density of 1279.0 kg/m^3^ was determined experimentally, the bend strength was 37.9 MPa, and information about tensile and shear strength were not declared by the manufacturer.

The second type of WPC cladding was material described in the text as WPC_60/30, which was 12 mm thick. The tested product was made of 60 wt % wood flour (hardwood, the exact type was not declared by the manufacturer), 30 wt % high-density polyethylene (referred to as HDPE) and 10 wt % additives [39]. Its bend strength was 21.7 MPa and tensile and shear strength were not declared by the manufacturer. The material density was 1210 kg/m^3^, however, a density of 1230 kg/m^3^ was determined experimentally.

The last tested material, referred to as WPC_50/38, was 9 mm thick and made of 50 wt % poplar wood flour, 38 wt % HDPE and 12 wt % additives (light stabilizer, coupling agent, anti-ageing component, UV retardant and colorant) [40]. The bend strength was 15.0–17.0 MPa, tensile strength around 4.9 MPa and shear strength of 2.2 MPa. A material density of 1250 kg/m^3^ was determined experimentally. Some product sheets did not contain information about material density. However, as Klyosov verified [4], the ratio of additives, in particular that of coupling agents, may significantly increase material density, and most importantly, it affects the material’s ability to absorb liquid. Specification of additives was provided only by one manufacturer, that is why the volumetric density was determined. The higher the volumetric mass density of WPC, the worse the wettability of the surface may be expected. 

### 2.2. Contact Angle Measurement

Prior to the manufacturing of the test samples it was necessary to determine the wettability of the surface of the selected materials. For all the selected materials a simple wettability test was performed according to ČSN EN 828 (it is the national equivalent to EN 828:2013. The wettability test was also repeated after the application of the chosen surface modification. 10 drops of distilled water were placed on the surface of the material. The technical standard for water recommends a volume of 2–6 μL [41], a volume of 3 μL was used.

### 2.3. Surface Treatment

#### 2.3.1. Mechanical Treatment

The mechanical modification of bonded surfaces was performed using P40 grid sandpaper. A layer of c. (0.25 ± 0.10) mm thickness was removed from the test samples. The thickness of the layer that was removed was determined using a 150 mm XTline P13430 digital Vernier caliper (XTline s.r.o., Velké Meziříčí, Czech Republic) with a rated accuracy of 0.01 mm and measured at three points. Microscopic examination of surface topography was not performed. All test samples were roughened by the same person to avoid any distortion of the results.

#### 2.3.2. Chemical Treatment

The ratio of components was determined based on the calculations of mass concentration of the solution, 10 g of 98% NaOH and 88 g of distilled water were used to achieve the desired solution concentration. The quantity was measured using digital scales with an accuracy of 0.01 g. The surface of the adherend was covered with the solution for c. 3 min. Subsequently, the surface was dried with blotting paper.

#### 2.3.3. Physical Treatment

**Figure 3:**
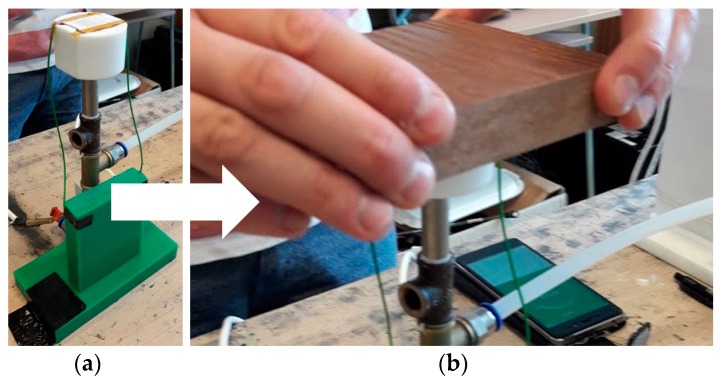
Plasma surface treatment: (**a**) Portable source of Multi-Hollow Surface Dielectric Barrier Discharge plasma; (**b**) Surface Treatment of the test sample (here with WPC_45/45).

The MHSDBD generated atmospheric-pressure plasma on a surface area of 18 × 18.9 mm. The contactless testing mode was used in this experiment. The samples were held by hand and always treated in the central part of the MHSDBD by gentle movements, see Figure 3. Plasma exposure time for all types of WPCs was 10 s. The power input of the MHSDBD during operation was 30 W in a flow air mode of 8.0 l/min.

### 2.4. Specimen Preparation

All WPCs were supplied in the form of planks, the original dimensions of which had to be adjusted to suit the requirements of the selected testing methods. The two most common destructive test methods were selected. The joints were tested for tensile and shear strength. For testing the adhesion of bonded joints at tensile stress, recommendations given in the ČSN 73 2577 standard were respected (the national standard describes steps similar to pull-off adhesion testing). All samples consisted of two components—the chosen façade cladding and the load-bearing substructure. The cladding was cut into squares of side length l/b = 100 mm, and the load-bearing substructure was represented by an aluminium disc with a circular cross-section area of A_ef_ = 2 500 mm^2^ [42]. The dimensions recommended in the ČSN EN 1465 standard [43] were used for test samples to determine the shear strength of a single lap joint under tensile stress. The test specimens were again made of two components, both with the dimensions b/b_ef_ = 25 mm and l_1_/l_2_ = 100 mm. One component represented the load-bearing substructure, the other the façade cladding. A standardized design of a single lap joint sample was used with l_ef_ = 12.5 mm.

The recommended thickness of the adhesive joint was 3 mm. To avoid influencing the results negatively, this parameter was strictly followed since it is general knowledge that the thickness of the adhesive layer has a significant effect on the resulting mechanical properties of the bonded joint [24,27,44,45,46,47,48,49].

When a physical or chemical treatment was applied to WPC surfaces, a cleaning agent was used before the surface treatment. Where mechanical treatment was applied, surfaces were cleaned after the roughening to remove all debris. To one half of the test samples (i.e., 5 samples) primer was subsequently applied. On the second half of the samples, the primer was not applied. This procedure would determine the effectiveness of the selected surface modifications as well as the effect of the primer on the joint’s efficiency and strength. The test samples were kept in a standard, dry and clean environment, at constant temperature (23 ± 2) °C and humidity (55 ± 10) % for 28 days, and left to cure statically.

### 2.5. Strength Test

**Figure 4:**
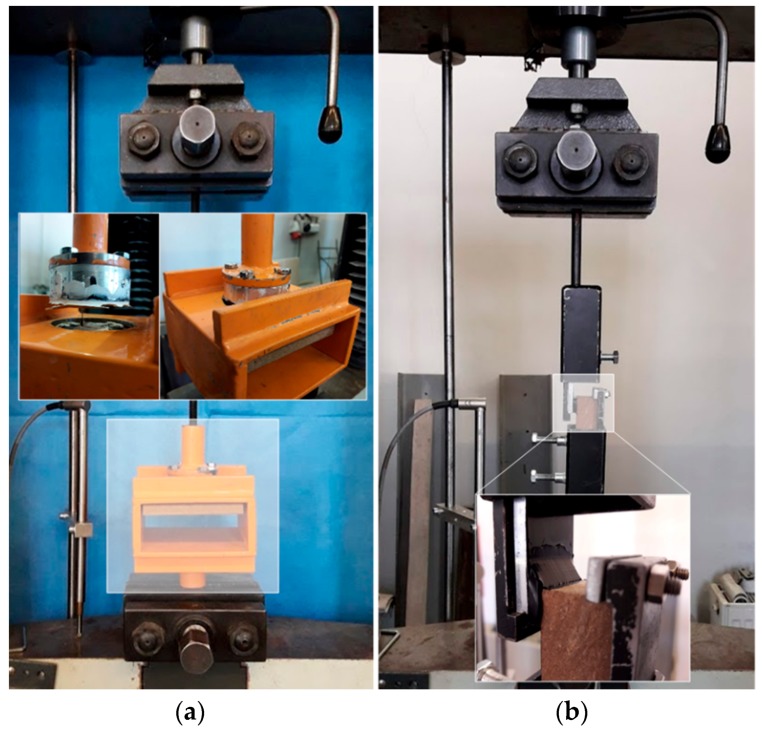
Test setup: (**a**) tensile test; (**b**) shear test.

A Heckert FP 10/1 tearing machine (ZwickRoell LP, Kennesaw, GA, USA) was used to record the development of deformations in the tested joints in relation to the applied load and time. The testing range of the machine is from 0 to 10 kN. Axial load was always applied on the samples. For this purpose molds were designed and made for the samples to be attached to the jaws of the tearing machine. The strain rate was set at 5 mm/min. The displacement of the test samples was recorded using a HBM 1-WA/100 MM-T inductive sensor (with a maximum deviation of 0.15%, Hottinger Baldwin Messtechnik GmbH, Darmstadt, Germany), which was placed on the cross member of the tearing machine, see the test setup in Figure 4.

An HBM Spider8 measuring station and catman^®^easy (V2.1) software (both Hottinger Baldwin Messtechnik GmbH, Darmstadt, Germany) were used for data recording. The load and joint elongation were recorded at a 5 Hz data storage frequency. The tests were carried out at a temperature of (20 ± 5) °C and relative humidity of (50 ± 20)%.

### 2.6. Data Analysis

Contact angles were determined by capturing images of water drops and evaluating them using ImageJ software and the Contact Angle extension module. The output of this simple testing method was the determination of the arithmetic average of the contact angles α and of the wettability of the selected surfaces. 

The failure modes of test samples were also evaluated. A scale based on recommendations presented in the ČSN ISO 10365 standard [50] (the national equivalent to ISO 10365) and in the international technical standard ASTM D 5573 [51] was prepared. For all tested combinations of joints, the predominant failure mode was determined.

Based on the recommendations of the aforementioned standards, the tensile stress (σ_adh_ in MPa) and shear strength of a bonded SLJ under tensile stress (τ in MPa) was determined. For each selected set of measured and calculated values, an arithmetic average was calculated, together with standard deviation. Subsequently, the coefficient of variation was calculated using the given values. The values of the coefficient over 20% indicate an ineffective surface treatment method. The elongation of the bonded joints was continuously recorded during the tests. Using the recorded values, it was possible to calculate the tensibility (δ in %) of a bonded joint. As only the change in length was monitored during the tests, we can further talk about relative elongation. The tensibility is the expression of the relative elongation in percentages.

## 3. Results and Discussion

### 3.1. Contact Angle Measurement

The results presented in Table 1 show that it is not always possible to improve wettability using surface modification. Based on these results, it can be assumed that the chosen mechanical surface modification will be the least appropriate option for improving the adhesive properties, however, Oporto et al. [18] monitored similar results. They examined a 20% decrease of surface energy after mechanical surface modification. Nevertheless, they observed a 60% and 80% increase in shear strength. The contact angle measured after physical and chemical modification showed more than a 40% improvement in surface wettability.

### 3.2. Failure Mode

The purpose of this assessment was to determine the predominant type of failure mode for each set of test samples and, if possible, to determine whether the selected method of surface modification has any effect on the failure mode of the bonded joint. To fully understand the adhesive properties of bonded surfaces, a description of their failure modes is necessary, as even under the same testing conditions, the same stress rate and with the application of the same adhesive, entirely different failure modes may occur in various materials.

The results presented in Table 2 and Table 3 show that only three types of failure occurred: AF–adhesive failure; SF–substrate (adherend) failure and NF–the sample was not broken. 

Adhesive failure occurred in almost all cases, although, in some combinations very high joint strength was observed. The monitored failure modes to some extent also disproved hypothesis that suitable surface treatment might compensate for primer usage. The adhesive failure mode suggests poor bondability of the bonded surface. In practice, this type of failure would pose considerable danger if it occurred on an actual façade. While cohesive failure occurs gradually, adhesive failure and the subsequent fall of the cladding is often very fast.

**Figure 5:**
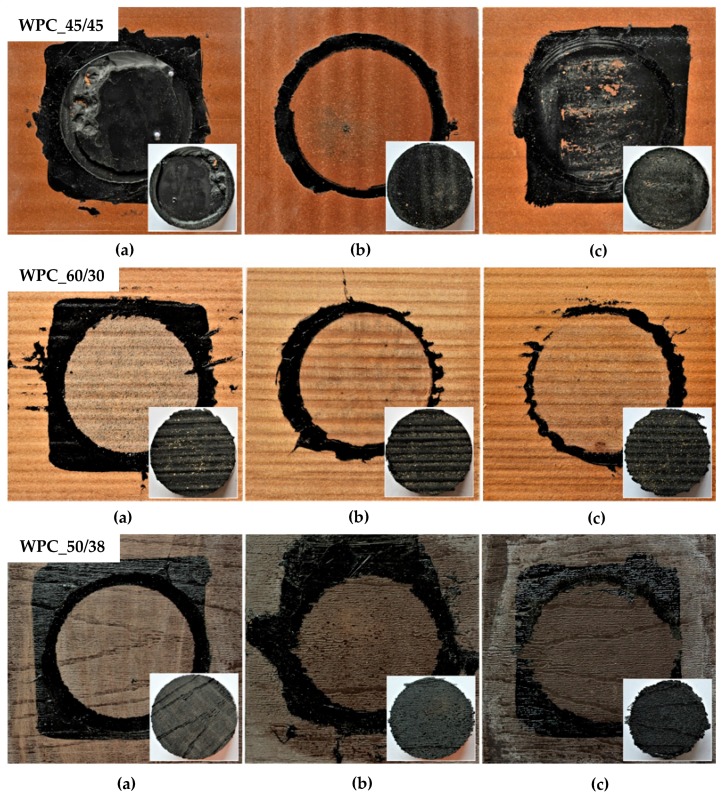
Typical failure mode of test samples after adhesion (tensile) test: WPC_45/45 (**a**) sample with primer, plasma treatment (86% AF; 14% CF); (**b**) sample without primer, chemical treatment (100% AF); (**c**) sample with primer, chemical treatment (100% AF); WPC_60/30 (**a**) sample with primer, chemical treatment (100% AF); (**b**) sample without primer, mechanical treatment (100% AF); (**c**) sample without primer, chemical treatment (100% AF); WPC_50/38 (**a**) sample with primer, mechanical treatment (100% AF); (**b**) sample without primer, plasma treatment (100% AF); (**c**) sample with primer chemical treatment (100% AF).

In combination with the WPC_60/30, only the adhesive failure mode was observed. Even though this failure of the bonded joint was dominant, the deterioration of the bonded surface was detected in all examined cases and wood fibers were visible, see the example in Figure 5 (WPC_60/30). This result is similar to the conclusion presented by Oushabi et al. [32], who stated that 10 wt % NaOH surface treatment resulted in surface damage and fiber degradation. In the presented case, due to this damage, slight penetration of the primer into the modified surface was observed. In Figure 5 (WPC_60/30 a)), the effect of chemical surface treatment is visible. All observed adhesive failures occurred in the interface between the primer and the WPC cladding. In combination with WPC_45/45, other types of failure than adhesive failure of the joint were also observed. In a few cases, the bonded joint was not broken, even though the limit of the tearing machine was reached. In several cases, substrate failure occurred. When multiple failure modes occurred, see example in Figure 5 (WPC_45/45 a)), the failure mode was classified according to the prevalent mode, in the example case it was the adhesion failure mode.

**Figure 6:**
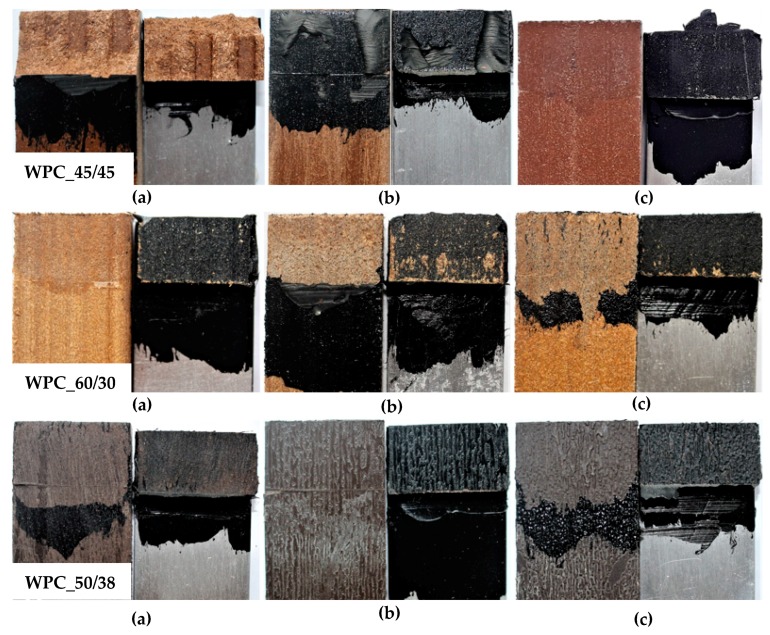
Typical failure mode of test samples after shear test: WPC_45/45 (**a**) sample with primer, plasma treatment (100% SF); (**b**) sample with primer, mechanical treatment (74% AF; 26% CF); (**c**) sample without primer, chemical treatment (100% AF); WPC_60/30 (**a**) sample without primer, plasma treatment (100% AF); (**b**) sample with primer, mechanical treatment (100% AF); (**c**) sample with primer, chemical treatment (100% AF); WPC_50/38 (**a**) sample with primer, mechanical treatment (100% AF); (**b**) sample without primer, chemical treatment (100% AF); (**c**) sample with primer plasma treatment (100% AF).

Similar results were observed after the shear test. However, the more significant effect of chemical surface modification was monitored, as shown in Figure 6. In combination with WPC_45/45, SF was witnessed after the physical and chemical surface modification, see Figure 6 (WPC_45/45 a)). Adhesive failure was observed in all tested combinations with WPC_60/30 and WPC_50/38. Again, the effect of chemical treatment was monitored predominantly in combination with WPC_60/30, however, it did not affect the failure mode. Moreover, slight surface deterioration of WPC_60/30 could be seen in all presented examples after the application of all surface treatments. 

The improvement of adhesive properties was observed in all tested samples with PVC-based WPC, however, the effect was not so prominent in combination with PE-based WPCs. Moreover, although changes were observed in all samples after surface modification, these were not confirmed by failure mode evaluation. The combination of the embossed surface and the HDPE component resulted in both small changes in the surface adhesion and almost 100% occurrence of adhesive failure. The prevalence of the given failure mode in the test samples was ambiguous, since all tested samples had a visibly damaged surface.

The selected surface modifications were completely unsuitable in terms of improving the adhesion properties of WPC_50/38. The poor results can be most likely attributed to the silicon layer (lubricant) that is protecting the surface of the material and polyethylene component. The surface of PVC-based WPC (i.e., WPC_45/45) was modified by all tested treatments. This cladding was the only sample containing this type of thermoplastic and at the same time was the only cladding the treated surface of which was not originally deeply embossed. This allowed the modification of the bonded area to be done more evenly. The deeper surface embossment of PE-based WPCs prevented an even application of surface modifications, and the insufficient surface adhesion in these areas caused premature joint failure.

### 3.3. Tensile/Shear Stress and Tensibility of Bonded Assemblies

All test samples without surface treatment debonded during the curing period; therefore, only the samples with a treated bonded area were tested and evaluated. 

Based on the results of the contact angle measurements and the determination of surface wettability, it can be assumed that all samples with a mechanically modified surface would disintegrate before even being tested. However, this hypothesis was not confirmed by any of the tested combinations. Not even in the case of samples without primer.

In combination with WPC_50/38, the best results were achieved after mechanical surface treatment, even though the bond strength was very small compared to the other tested combinations, see Table 4 and Table 5. The results achieved with the WPC_50/38 cladding were the worst of all the tested combinations. It was observed that this cladding had a very thick protective silicone-based layer (lubricant), compared to the other WPCs, which protects the surface against any damage after its extrusion. None of the tested surface modifications removed a sufficient thickness of this layer; therefore, any significant improvement in adhesive properties was not monitored. A similar observation was reported by Oporto et al. [18]. 

**Figure 7:**
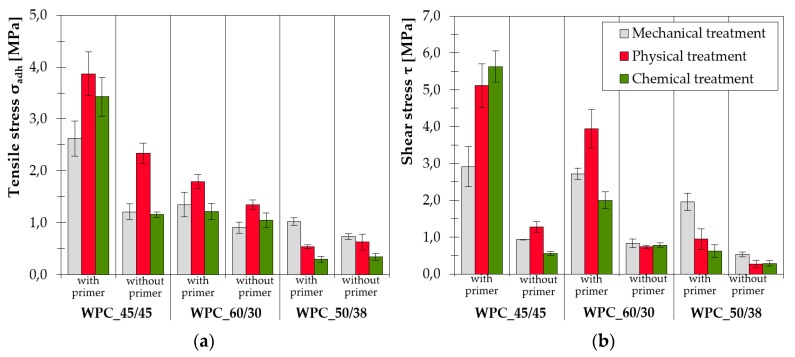
Comparison of average stress values: (**a**) in tension and (**b**) in shear after surface treatment. The results of samples with and without primer are compared. The standard error in the mean is presented in the error bar.

In some cases with WPC_45/45 no failure was recorded as the loading limit of the testing machine was exceeded. This referred to the samples with primer and plasma surface modification. The bonded joint was very durable even if the primer coating was not applied. The recorded strength as well as maximum joint elongation at break were diametrically different compared to other tested WPCs. It is also the only sample of the composite that was composed of PVC, the other two types of cladding contained HDPE. This fact influenced the effectiveness of the selected surface modifications. The comparison of results presented in Figure 7 shows that MHSDBD plasma treatment was also the most effective method in combination with WPC_60/30.

The results of the tests determining the shear strength of SLJs under tensile stress are clearer in showing that using all the components of a facade mounting system is essential. Figure 7b shows that the differences between the samples with and without primer are diametrical. Although the bonded area was sufficiently modified in all examined alternatives, there was a significant increase in strength in the test samples coated with primer.

**Figure 8:**
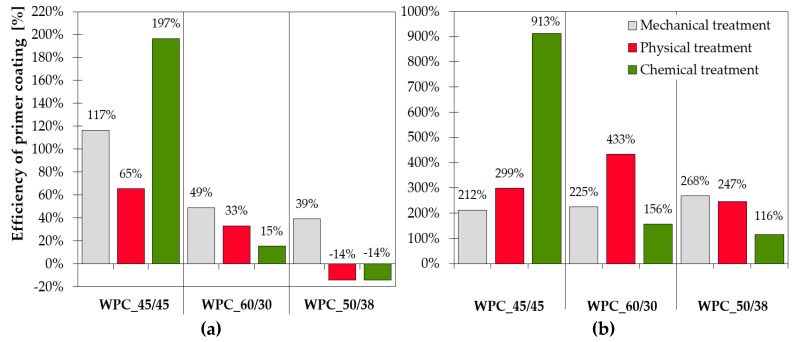
Comparison of surface treatment efficiency: (**a**) tensile test and (**b**) shear test. The higher the percentage is the more important the application of primer coating and the less effective the surface treatment. The negative values indicate that the results of samples without primer were better.

The effect of the primer and increase in bond strength is clear from the comparison presented in Figure 8. The difference in joint strength was compared. The higher the percentage value the more important the primer coating is to achieve an efficient bond. The negative values presented in Figure 8a in combination with WPC_50/38 indicate that samples without primer achieved better results. The application of primer did not affect the bond tensile strength and efficiency. The negative values also indicated that the surface wettability was not increased by the modification.

Comparable results were achieved by physical modification. The MHSDBD plasma treatment appears to be the most suitable option for WPC_45/45 as well as for WPC_60/30. A different exposure period might have improved the adhesive properties of the surface even more. On the other hand, it seems to be that the selected chemical surface treatment was the least effective.

The importance and necessity of using all the components of the mounting system is obvious. The measured values of the strength of the bonded test samples on which primer was not used are too low, and the differences in comparison with the samples containing the primer are quite ambiguous. Nevertheless, some isolated results show that the choice of an appropriate surface modification may increase the effectiveness of a bonded joint even without using the primer coating, and this is certainly a good direction to follow in the future.

**Figure 9:**
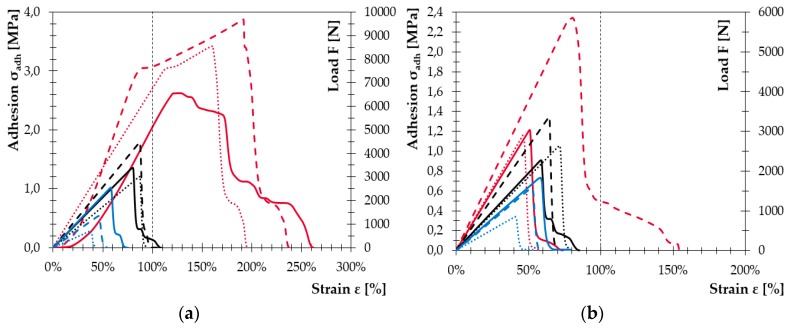
Comparison of average stress–strain curves from tensile test: (**a**) samples with primer and (**b**) samples without primer. Color marking: RED—WPC_45/45; BLACK—WPC_60/30; BLUE—WPC_58/30. Solid lines are samples with mechanical treatment, dashed lines are samples with physical treatment and dotted lines are samples with chemical treatment.

**Figure 10:**
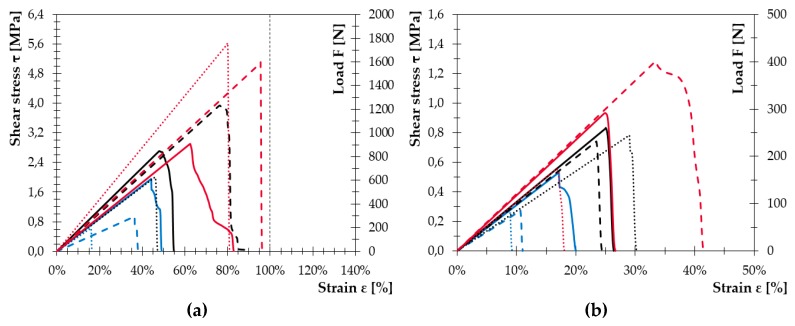
Comparison of average stress–strain curves from shear test: (**a**) samples with primer and (**b**) samples without primer. Color marking: RED—WPC_45/45; BLACK—WPC_60/30; BLUE—WPC_58/30. Solid lines are samples with mechanical treatment, dashed lines are samples with physical treatment and dotted lines are samples with chemical treatment.

While for the samples with WPC_45/45, the selected modifications were effective in both testing methods since high ultimate stress values were achieved; in the case of WPC_60/30 and WPC_50/38, the effect of surface modification was not so clear. Moreover, the ‘ideal’ deformation of the bonded joint during the tests occurred only in combination with WPC_45/45, when the bonded joint transformed progressively from elastic deformation to plastic deformation and the disintegration of the joint appeared only after the cohesive or substrate failure. In other cases, this phenomenon was rare, as depicted in Figure 9. The average stress-strain curves of all tested samples shown in Figure 9 (samples after tensile test) and in Figure 10 (samples after shear test), present the effectiveness of physical treatment. The MHSDBD plasma source successfully modified bonded surfaces of all tested types of WPCs. The tensile strength (adhesion) of plasma treated samples increased by 100% compared to the untreated samples even when primer was not used.

## 4. Conclusions

In this study, a moisture-curing permanently elastic one-component polyurethane pre-polymer adhesive was used to study different surface treatments of bonded areas and its effect on the strength of the adhesive joint. The main purpose was to evaluate the effect of three different surface treatment methods: A mechanical, a physical and a chemical treatment, on the adhesive properties of WPCs façade cladding. Three different formulations of WPC were tested: one PVC-based and two PE-based (HDPE) WPCs. Two destructive standardized test methods were selected to provide sufficiently detailed information for the analysis of the effect of surface treatment on the adhesive properties of the WPC cladding:

- An improvement in the adhesive properties was demonstrated by the increase in the strength of the bonded joint after tensile as well as shear tests. In one combination, the achieved strength was nearing the maximum strength of the adhesive system declared by the manufacturer.
- A positive effect of surface modifications on the failure mode was observed. In two combinations substrate failure was the predominant failure mode after the tensile test.
- The selected surface treatments were more suitable for PVC-based WPCs than for PE-based WPCs. Furthermore, it was confirmed that the effect of the thermoplastic component of WPC on its adhesion properties is dominant.
- The effect of the wood fibers in WPCs was minor in all tested combinations. The assumption that a higher content of wood flour would have a positive effect on the adhesive properties was not confirmed.
- The most consistent results were achieved after physical modification of the bonded surface. However, different exposure period for each tested material seems to be necessary. Physical modification using the MHSDBD plasma source can be considered universal surface treatment for the selected WPCs. It is also the cleanest and the least time-consuming method.
- The presented results confirm the well proven fact that the adhesive properties of the surface of WPC materials are very poor, and without modification of the bonded surfaces, their bonding is almost impossible, or rather the effectiveness of the joint is negligible.

The tests have shown that selected adhesive/mounting system is suitable for the bonding of WPCs cladding, however, prior verification of the compatibility of selected materials is required. This conclusion has proved to be decisive in terms of the statement presented in the introduction, i.e., that adhesive-bonded joints are more suitable alternative than traditional mechanical joints. The necessity to conduct a detailed and often financially demanding experimental assessment of the compatibility of selected materials, frequently with unsatisfying results, is a difficult obstacle to overcome when arguing e.g., with investors and/or clients about why they should choose adhesive bonding to anchor WPCs cladding. For these reasons, the plasma treatment is in our opinion, the best surface modification method for WPCs.

## Figures and Tables

**Table 1:** Average contact angle α of WPC surfaces WITHOUT and WITH surface treatment ^1^.

Surface Treatment/WPC Type	WPC_45/45 [°]	WPC_60/30 [°]	WPC_50/38 [°]
Without Treatment	90.94 ± 2.96	80.60 ± 4.29	98.89 ± 2.97
Mechanical (P40)	104.95 ± 2.26	103.83 ± 1.47	118.77 ± 1.28
Physical (MHSDBD)	63.51 ± 2.15	50.99 ± 2.61	45.29 ± 1.09
Chemical (10% NaOH)	67.98 ± 3.55	67.43 ± 3.03	67.13 ± 1.94

^1^ The average values are a summary of ten measurements conducted for each WPC cladding before and after surface modification.

**Table 2:** Predominant failure mode of samples WITH and WITHOUT primer after tensile test ^1^.

Surface Treatment/ WPC Type	WPC_45/45		WPC_60/30		WPC_50/38	
	With	Without	With	Without	With	Without
Without Treatment	AF 100%	AF 100%	AF 100%	AF 100%	AF 100%	AF 100%
Mechanical (P40)	SF 80%	AF 100%	AF 100%	AF 100%	AF 100%	AF 100%
Physical (MHSDBD)	NF 60%	AF 100%	AF 100%	AF 100%	AF 100%	AF 100%
Chemical (10% NaOH)	AF 80%	AF 100%	AF 100%	AF 100%	AF 100%	AF 100%

^1^ The predominant failure mode was determined for one set of samples (i.e., 5 samples with or without primer).

**Table 3:** Predominant failure mode of samples WITH and WITHOUT primer after shear test ^1^.

Surface Treatment/ WPC Type	WPC_45/45		WPC_60/30		WPC_50/38	
	With	Without	With	Without	With	Without
Without treatment	AF 100%	AF 100%	AF 100%	AF 100%	AF 100%	AF 100%
Mechanical (P40)	AF 80%	AF 100%	AF 100%	AF 100%	AF 100%	AF 100%
Physical (MHSDBD)	SF 100%	AF 100%	AF 80%	AF 100%	AF 100%	AF 100%
Chemical (10% NaOH)	SF 80%	AF 100%	AF 100%	AF 100%	AF 100%	AF 100%

^1^ The predominant failure mode was determined for one set of samples (i.e., 5 samples with or without primer).

**Table 4:** Average tensile stress σ_adh_ (in MPa), variation coefficient (in %) and tensibility (in %) of samples WITH and WITHOUT primer ^1^.

WPC Type/ Surface Treatment		Mechanical (P40)		Physical (MHSDBD)		Chemical (10% NaOH)	
		With	Without	With	Without	With	Without
**WPC_45/45**	σ_adh_	2.62	1.21	3.87	2.34	3.43	1.16
	VC ^2^	12.99	12.34	10.87	8.34	10.94	4.44
	δ	127.52	51.21	191.67	79.79	160.58	46.27
**WPC_60/30**	σ_adh_	1.35	0.91	1.79	1.35	1.21	1.05
	VC	17.79	11.45	7.41	6.81	13.10	13.26
	δ	87.67	59.17	87.54	64.54	87.67	71.63
**WPC_50/38**	σ_adh_	1.02	0.73	0.54	0.63	0.29	0.34
	VC	8.08	8.08	6.88	24.06	19.22	19.65
	δ	58.40	58.52	45.81	52.60	38.63	40.98

^1^ The average values are a summary of five measurements conducted for each tested combination.

^2^ VC is an abbreviation of variation coefficient.

**Table 5:** Average shear stress τ (in MPa), variation coefficient (in %) and tensibility (in %) of samples WITH and WITHOUT primer ^1^.

WPC Type/ Surface Treatment		Mechanical (P40)		Physical (MHSDBD)		Chemical (10% NaOH)	
		With	Without	With	Without	With	Without
**WPC_45/45**	τ	2.91	0.93	5.11	1.21	5.63	0.56
	VC ^2^	18.58	0.78	11.68	11.27	7.54	8.70
	δ	62.41	24.95	95.38	33.46	79.93	16.76
**WPC_60/30**	τ	2.71	0.83	3.94	0.74	2.00	0.78
	VC	5.80	14.54	13.25	4.77	11.44	7.60
	δ	47.92	25.04	76.44	23.44	46.24	29.01
**WPC_50/38**	τ	1.95	0.53	0.95	0.27	0.62	0.29
	VC	12.07	12.73	29.77	38.39	27.14	24.23
	δ	44.29	17.11	36.31	10.70	15.99	8.88

^1^ The average values are a summary of five measurements conducted for each tested combination.

^2^ VC is an abbreviation of variation coefficient.
